# Fungal ethnoecology: observed habitat preferences and the perception of changes in fungal abundance by mushroom collectors in Poland

**DOI:** 10.1186/s13002-021-00456-x

**Published:** 2021-04-21

**Authors:** Marcin Andrzej Kotowski, Zsolt Molnár, Łukasz Łuczaj

**Affiliations:** 1 Institute of Ecology and Botany, Centre for Ecological Research, Vácrátót, H-2163 Hungary; 2 Botanical Garden Center for Biological Diversity Conservation in Powsin, Polish Academy of Sciences, Warsaw, Poland; 3 Department of Botany, Faculty of Biotechnology, University of Rzeszów, Pigonia 1, 35-310 Rzeszów, Poland

**Keywords:** Ethnomycology, Ethnoecology, Folk habitats, Perception of change, Macromycetes, Fungi, Abundance, Habitat preference, Hypotheses, Mazovia

## Abstract

**Background:**

Scientists frequently raise the topic of data deficiency related to the abundance and distribution of macrofungi in the context of climate change. Our study is the first detailed documentation on locals’ perception of fungal ecology which covers a large mycophilous region of Europe (Mazovia, Poland).

**Methods:**

A total of 695 semi-structured interviews were carried out among local informants in 38 localities proportionally distributed throughout the study area (one locality approximately every 30 km). Interview questions concerned fungi species collected, their perceived habitats, and whether any changes had been noted in their abundance. As many as 556 respondents provided information concerning fungal ecology. In these descriptions, 35 taxa were mentioned by at least 5 respondents.

**Results:**

The data collected during interviews allowed us to create collective folk descriptions of habitat preferences and a list of 98 different macro-, meso-, and microhabitats of macrofungi described by the respondents. This list of recurring habitats assigned to particular macrofungal taxa coincides with, and sometimes exceeds, data available in scientific publications. Some habitat preferences observed by the informants have not yet been researched or tested by science.

Out of 695 respondents, 366 (53%) noticed a steady decrease in local macrofungi abundance, and only one person claimed to have observed a steady increase. *Imleria badia* was the only species with increased abundance, as noted by fifteen independent respondents. The main listed reason for abundance decrease was drought (*f* = 186).

**Conclusions:**

Collected information on the ecology of fungi shows that local knowledge does not generally diverge from scientific knowledge. The acquired information related to macrofungal abundance and ecology may also be used as a tool for the formulation of new scientific questions and theories. The analysis of local fungi observations might contribute to broadening knowledge about local changes in fungi and enable new estimations related to large-scale analysis of macrofungal abundance.

## Introduction

Since the mid-1950s, scientists have explored patterns of shared environmental knowledge that emerged from indigenous practices based on local human-nature relationships [1, 2]. This new research area came to form the broad cross-discipline of ethnoscience—a field of science based on collaboration between social and natural sciences [3]. Researchers who study local ecological issues have noticed that local traditional communities have developed an extensive body of traditional ecological knowledge (TEK) about plants, animals, fungi, ecosystems, landscapes, and the processes and changes they undergo [4]. This knowledge emerged from long-term observations, experiments, and direct personal interactions with surrounding living nature [5]. A rise in scientific interest in this body of knowledge led to the development of ethnoecology—a new sub-field of ethnoscience. Ethnoecology is the scientific study of how different groups of people living in different locations understand the ecosystems around them and what relationships they form with their surrounding environments [6]. Ethnomycology broadly considers human engagement with the kingdom of fungi, bringing together the interests of the humanities, fine arts, and social and natural sciences [7]. Our present research was conducted following a traditional view on fungal ecology.

Traditional ecological knowledge is not only ‘used’ by the local communities that develop and possess this knowledge, but it also provides its users with a deep understanding of the status and changes to the local environment. This knowledge can often complement scientific understanding [8], help environmental monitoring [9], and support the planning and execution of adaptive conservation management [10]. Additionally, local and traditional ecological knowledge can help to develop new scientific questions and testable hypotheses [11, 12]

Traditional ecological knowledge can be related to habitat and ecosystem types, including habitat classification and landscape partitioning [13, 14]. Although this domain still requires research, recent studies analysing folk habitat types have proven the complexity and multidimensional characteristics of folk habitat descriptions and landscape partitionings. The studies conducted by Babai and Molnár [15] among Csángó people living in Gyimes (Carpathians, Romania) have also underlined the importance of the scale dimension, which plays a major role in folk habitat classifications. The significance of topographical and topological aspects of scale in folk habitat classifications has also been confirmed by Gantuya et al. [16] among Mongolian herders. In general, folk habitat types can be grouped into macro-, meso-, and micro-scale habitats. Macrohabitats usually occupy large areas and comprise many habitat types, forming a mosaic. Mesohabitats are usually smaller in extension, homogenous, and often dominated by a single type of vegetation. Microhabitats are embedded in mesohabitats and provide special niches for particular species [14].

Because environmental changes are caused not only by natural but also by societal processes, by interacting and shaping their environment, local communities have developed their own perception of these changes [17]. Recently, local observations of environmental change are becoming recognized by science [18]. According to Nakashima et al. [19], people who interact with nature on a daily basis display knowledge that can be essential in introducing measurements to adapt and fight climate change. In her work, Gantuya et al. [16], besides noticing the important role of seasonal changes and pasture dynamics in determining the most suitable grazing area, emphasized the importance of long-term ecological stability for local herders. Ujházy et al. [20] compared farmers’ and conservationists’ perception of landscape changes. The results showed that the two groups shared similar views on perceived landscape changes, but they evaluated these changes differently. Farmers mostly focused on the impact on habitat usefulness, while conservationists had a primarily eco-centric approach. The common message of studies focusing on folk knowledge in relation to environmental change is the need for a deeper understanding of local perceptions [18, 21]. Studying local knowledge could broaden our understanding of the trends in ongoing ecological changes [22]. Having completed quantitative analysis of a large number of interviews, it is also possible to provide important information on the heterogeneity of social landscape perception [20].

The few studies that document local and traditional knowledge of fungal habitats and population changes (incl. abundance) usually focus on individual species [23, 24]. Lampman [25], however, undertakes a complete documentation of fungi-related knowledge shared by the Tzeltal Maya of the Chiapas highlands. In his work, Lampman focuses on knowledge concerning wild edible fungi ecology. However, the characteristics he describes often only provide a general overview of locally used macrofungi, without any detailed data on particular species. Lampman recorded information on the relationship of particular taxa to characteristics such as substrate preference, but without providing quantitative data (e.g. number of informants).

In our present study on fungal ethnoecology, we have the following objectives:

- To document the habitat types used by local Polish mushroom collectors to describe the habitat preferences of various fungal taxa;
- To document the habitat preference of each mushroom species by appealing to the observations of a large number of mushroom collectors;
- To analyse local perception of macrofungal population trends (abundance) using local observations as a specific form of fungi monitoring;
- Finally, to generate a hypothesis for further research on fungi based on the above observations.

## Methods

### Research area

**Fig. 1:**
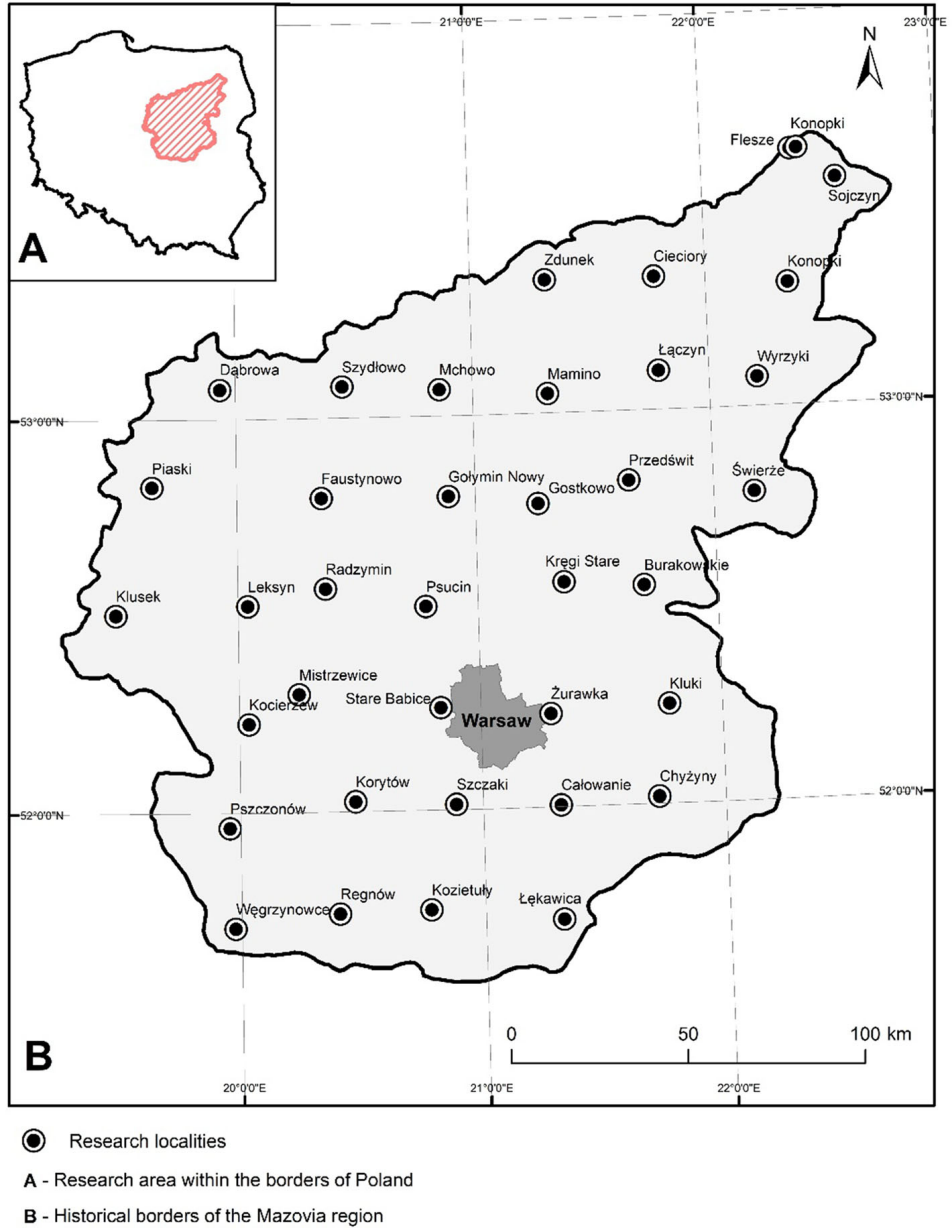
Research area

Mazovia is a historical region that lies in the Central-Eastern part of Poland. It is one of the ten major Polish historical regions within the country’s present-day borders. Mazovia was an independent principality throughout a major part of Polish mediaeval history [26]. In the case of the present study, its borders were determined by a map created for the ‘Historical Atlas of Poland in the 2^nd^ Half of the 16^th^ Century’ by Pałucki [27] (Fig. 1).

The region lies mainly within the current borders of the Mazovian Voivodeship and extends to part of the Łódź Voivodeship in the south-west and to Podlasie Voivodeship in the north-east. It covers about 33,900 km^2^, spreading over the Mazovian Lowland in the valleys of the Vistula, Bug, and Narew rivers. It is currently inhabited by around 5.03 million people [28]. Mazovia is characterized by a cold temperate climate with high annual temperature amplitudes and a transitional character between oceanic and continental [29]. The average temperature (VI–VIII) is around 18 °C in the summer and 1 °C during winter (XII–II). Average annual rainfall ranges from 550 to 600 mm [30]. Forest vegetation covers 23.3% of the research area [31], the majority of which are coniferous forests (64%) mainly composed of *Pinus sylvestris* L*.*. The other species that are the most abundant in mixed and deciduous forests are *Quercus robur* L*.* and *Betula pendula* Roth*.*

Folk cultural characteristics shared by people living in this historical region are currently difficult to find. However, the region is still inhabited by a few ethnographic groups. Usually, they can be distinguished by their local traditions and cultures. These groups are the Podlasianie, Mazurzy, Łowiczanie, and Kurpie [32]. The capital city of Warsaw is situated in the centre of Mazovia. Despite the broad urban sprawl surrounding Warsaw, there are even forests used for recreational mushroom picking in the city’s agglomeration.

The research was conducted in 38 villages or small market towns, which were dispersed in a 30-km grid throughout the whole Mazovian region (Fig. 1). These were Burakowskie, Całowanie, Chyżyny, Cieciory, Dąbrowa, Faustynowo, Flesze, Gostkowo, Kluki, Klusek, Kocierzew, Konopki (Grajewo County), Konopki (Łomża County), Korytów, Kozietuły, Kręgi, Leksyn, Łątczyn, Łękawica, Mamino, Mchowo, Mistrzewice, Nowy Gołymin, Piaski, Przedświt, Psucin, Pszczonów, Radzymin, Regnów, Sojczyn, Stare Babice, Szczaki, Szydłowo, Świerże, Węgrzynowice, Wyrzyki, Zdunek, and Żurawka (currently the district of Sulejówek).

This network of settlements forms part of the larger grid of the Ethnographic Atlas of Poland, which was also used to collect data on mushroom picking between 1964 and 1969. At that time, chosen localities were described as ‘large moderately backward’ settlements.

### Field research

The field research took place between 2014 and 2018, from April to November—the months of abundance of traditionally collected wild edible fungi in Poland. Data collection was spread evenly across the research period, while the volume of collected data depended mostly on weather conditions and population density. Data were collected through individual semi-structured interviews conducted among local informants, which constitutes the classic method in ethnobiology [33]. Aside from data concerning local knowledge on collected species, folk taxonomy and cultural significance presented in previous work [34], we have also documented knowledge about collected species ecology and their changes in abundance observed during the years of active fungi collection (usually since childhood to the day of interview). Information about macrofungi gathered or recognized as edible was collected by using the freelisting method. All freelists were made orally and written down. Questions relating to knowledge about species habitat and changes to abundance were asked in relation to each listed species. The information was acquired through informants’ answers to general questions: ‘Where would you look for this mushroom species?’, ‘Did you notice any changes in the abundance of this species?’, and ‘What do you think is the main cause of mushroom abundance changes?’ All of 695 respondents were asked questions concerning fungal habitat and abundance changes. Not everyone was able to answer them. In case of habitat descriptions, lack of answer was classified as ‘unknown’; therefore, it was not used in habitat description and analysis (Table 1). In case of abundance changes, lack of answer was classified as ‘unnoticed’ and is present in data analysis (Fig. 4).

**Table 1:** Habitat types used to describe the habitat preference of various mushroom species listed by the respondents (*n* = 556).

Habitat	Frequency	Habitat	Frequency
Pine *Pinus sylvestris* L. (occurrence correlated with pine presence)	1178	Blackberries (*Rubus* L. spp.)	8
Birch (*Betula* L. spp.)	746	Boar rooting (grounds disturbed by boar activity)	8
Mixed forests (coniferous and deciduous)	622	Orchards	8
Sandy soils	383	Water’s edge	8
Small/young trees	381	Firebreaks	7
Oak (*Quercus* L. spp).	345	Hazel (*Corylus avellana* L.)	7
Coniferous forests	334	On trees	7
Meadows	221	Hills/scarps	6
Moss (presence in the groundcover)	217	Potato fields	6
Terrain elevations/hillocks	206	Snow (under the snow-cover)	6
forest edge	170	Bogs	5
Deciduous forests	146	Near feeding rack	5
Various habitats (occurring in many unrelated habitats)	138	Stubble	5
Grasses	135	Thin forests	5
Old/tall forests	129	White moss (*Leucobryum glaucum* (Hedw.) Ångstr.)	5
Roadsides	104	Balks (i.e. strips between fields)	4
Tree stumps	103	Lichens	4
Common aspen	83	Parks	4
Humid soils	78	Short grass	4
Fields	76	Beech (*Fagus sylvatica* L.)	3
Trenches/depressions	62	Bird cherry (*Prunus padus* L.)	3
Litter with conifer needles	59	Black poplar (*Populus nigra* L.)	3
Thickets	52	Fallen pine bark/mulching bark	3
Cows/horses (presence – mainly pastures)	49	*Frangula alnus* Mill.	3
Spruce (*Picea abies* (L.) H.Karst.)	48	*Robinia pseudoacacia* L.	3
Forest clearings	43	Bog blueberry (*Vaccinium uliginosum* L.)	2
Alder (*Alnus* Mill. spp.)	41	Secondary forest	2
Heather (*Calluna vulgaris* (L.) Hull)	39	Near tree trunks	2
Under fallen branches	39	Poplars (*Populus* L. spp.)	2
Clear/light forest	35	Rich undergrowth	2
Clearcutting areas	33	Without undergrowth	2
Dry soils	33	Ash tree (*Fraxinus excelsior* L.)	1
Blueberries (*Vaccinium myrtillus* L.)	29	Burned areas	1
Open areas	29	Compost	1
Yards	28	Dense forest	2
High sun exposure	25	Elder trees (*Sambucus nigra* L.)	1
Fallows/wastelands	23	Elm (*Ulmus* L. spp.)	1
Among litter	21	Ferns	1
Hornbeam (*Carpinus betulus* L.)	19	Fertile soil	1
Juniper (*Juniperus communis* L.)	16	Fir (*Abies alba* Mill.)	1
Larch (*Larix decidua* Mill.)	15	Foxholes	1
Dead wood	14	Garbage dumps	1
Self-sown forest	14	Green moss	1
Forest plantations	12	Hardwood trees	1
Enshadowed areas	11	Near the bunkers (after the war)	1
Medium aged forests	10	Railroad trackway	1
Thick litter layer	10	Ridges	1
Behind the barn (buildings near open areas)	9	Thin litter layer	1
Animal manure	8	Near log piles	1

At least one landscape walk or joined collection trip was conducted in each village. The majority of voucher specimens for further identification were collected fresh during field interviews, and some were acquired in dried form from respondents. A total of 695 individual interviews have been conducted where respondents provided information on folk taxonomy of collected fungi species [34]. Among them, 556 respondents provided information on fungal ecology related to 92 taxa. Women accounted for 52% (362) and men for 48% (333). The age of informants ranged from 17 to 95. The mean age was 63 (SD = 13.7) and the median 64. Informants were selected during village walks or using the ‘snowball’ sampling technique [35]. The selection of informants was haphazard—based on their willingness to participate in the interview—and therefore socio-demographic characteristics were varied. However, like in most ethnobiological studies, we aimed at talking to middle-aged and older people.

### Data analysis

The majority of fungal fruiting bodies were identified with the support of mushroom pictures or identification guides [36]. Some of the interviews were conducted simultaneously with mushroom collection. This method enabled us to recognize taxa on the spot and to collect voucher specimens, which were additionally identified by DNA barcoding [34].

All folk habitat terms mentioned by the respondents in the interviews were extracted and grouped. Synonymous folk habitat names were grouped according to dimensions such as dominant symbiotic species, succession, land use, vegetation structure, forest vegetation physiognomy, geomorphology, soils, hydrology, human, and animal disturbances [15, 16].

**Fig. 2:**
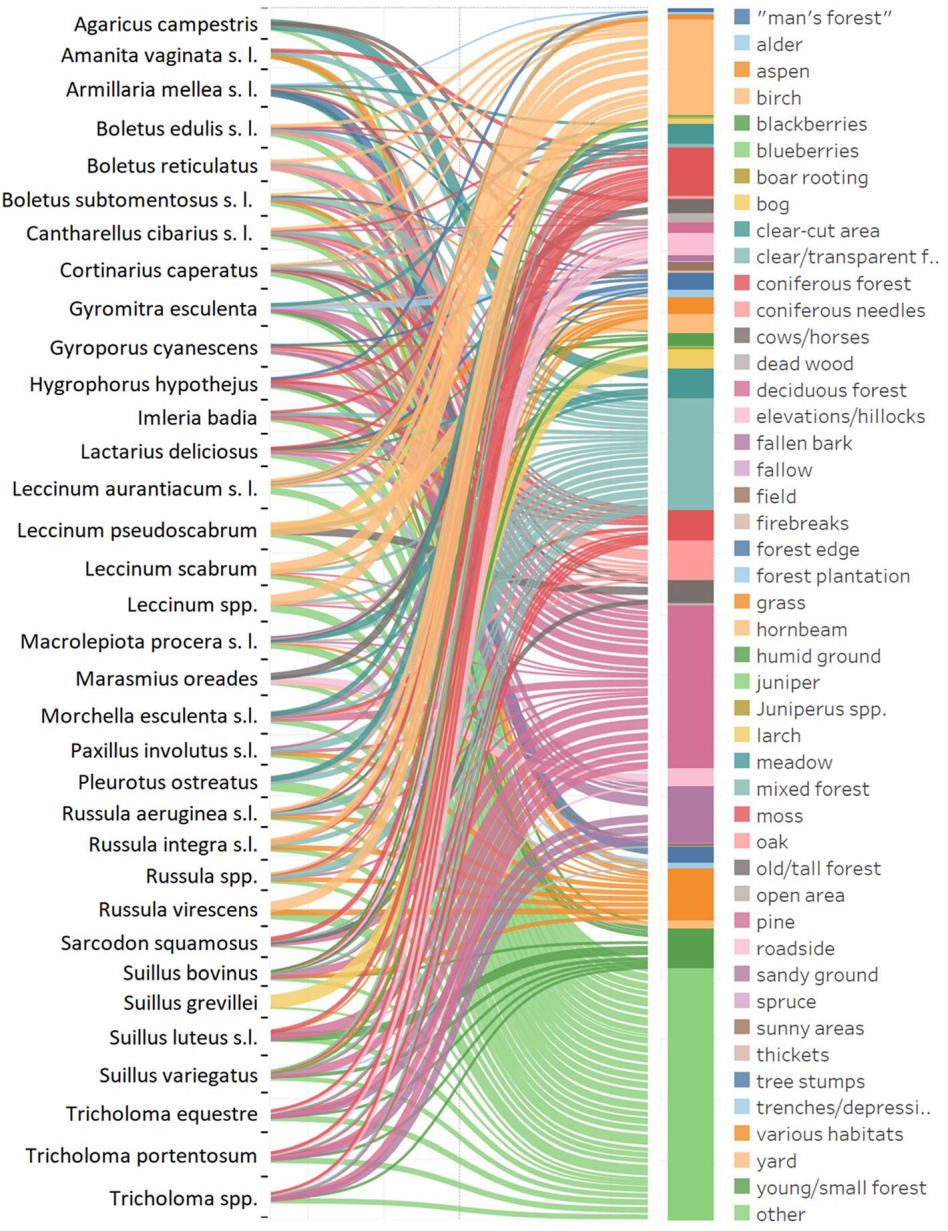
The main observed habitat types preferred by certain mushroom species according to local mushroom collectors in Mazovia, Poland. Colour shows details about habitat. Size of line indicates percentage of respondents mentioning certain habitat in a particular species description

After analysing 556 interviews and 3999 reports concerning particular fungal taxa, we also selected 35 taxa with 5 or more individual ecological descriptions (Fig. 2). In order to remove singular folk reports and focus on the most frequently mentioned habitats, we only selected habitats that were listed by more than 5% of respondents in relation to particular taxa and were listed more than once. Habitats mentioned by a fewer number of respondents were grouped as ‘other’. In order to present the acquired data, we used Sankey diagram created with the use of Tableau software version 2020.4.

**Fig. 3:**
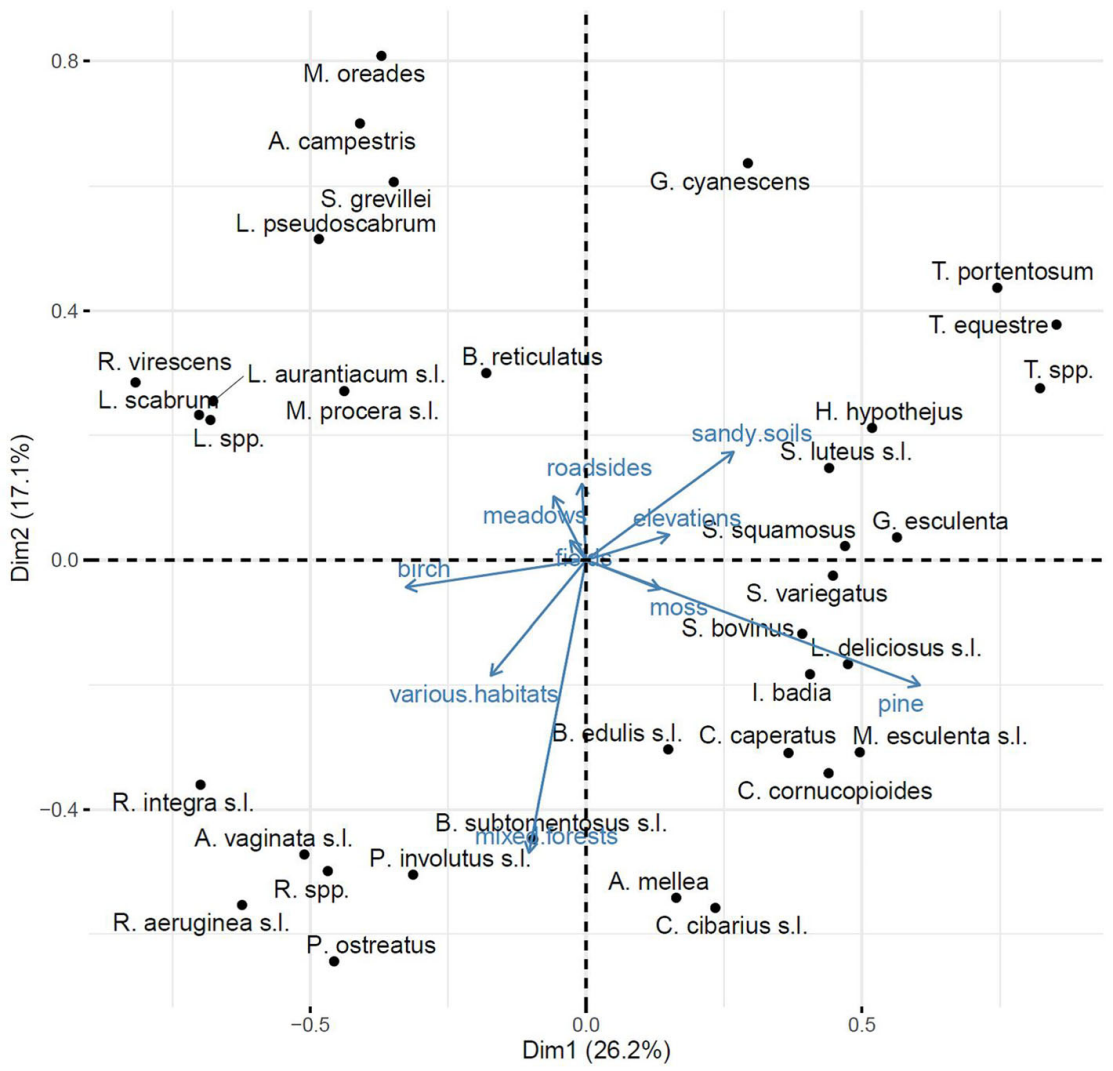
PCA of taxa in relation to habitats most frequently mentioned by the respondents

PCA analysis was conducted on the basis of the matrix of the most frequently mentioned habitats in relation to different fungal taxa, which were selected in analysis presented in Fig. 2. The main purpose of the principal component analysis is to compute the principal components (in this case elements describing fungal habitats) and use them to determine certain groups of species related to specific multidimentional habitat description. This allows for a reduction in the dimensionality of data while preserving its variation. The first principal components can define which direction maximizes the variation of projected points, therefore enabling the division of certain fungal species into groups with similar habitat preferences. Data processing included normalization using the min-max scaling method and singular value decomposition (SVD). PCA analysis was performed in R programming language using the FactoMineR package in Rstudio software [37] (Fig. 3).

In order to compare folk ecology descriptions with scientific knowledge, we used the ‘Checklist of Polish Larger Basidiomycetes’ [38] as a reference point for the *Basidiomycota* species and ‘Grzyby i ich oznaczanie’ [39] for Ascomycota. This was supplemented with data from other scientific publications.

**Fig. 4:**
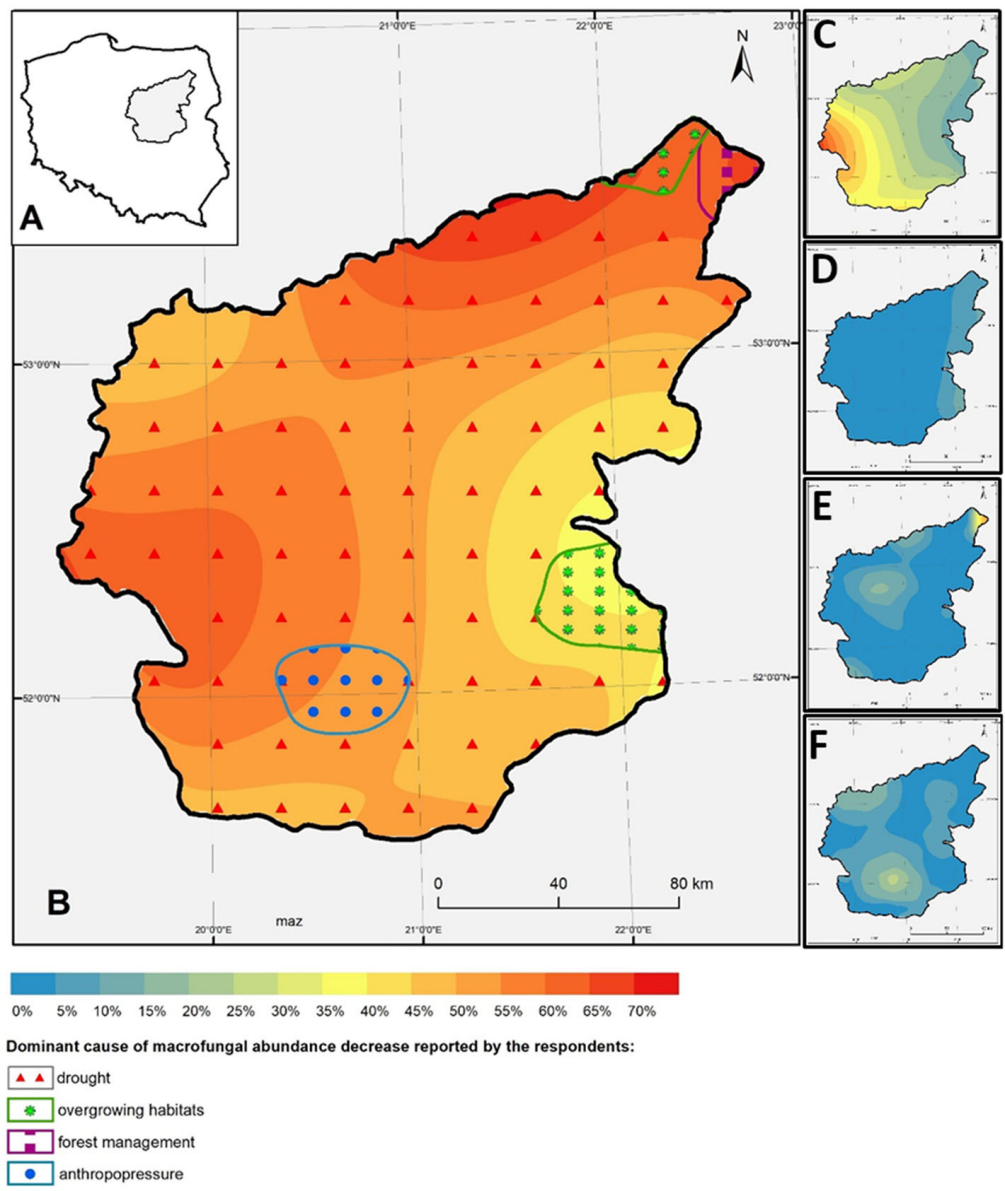
Percentage of residents who have noticed a steady decrease in edible macrofungi abundance (**a** research area within the borders of Poland; **b** historical borders of the Mazovia region and the percentage of respondents that have indicated a steady decrease of macrofungal abundance during lifelong observations; **c** percentage of respondents who reported drought as the main cause of fungal abundance decrease; **d** percentage of respondents who reported habitat overgrowth as the main cause of fungal abundance decrease; **e** percentage of respondents who reported forest management as the main cause of fungal abundance decrease; **d** percentage of respondents who reported anthropopressure as the main cause of fungal abundance decrease)

We recorded the number of respondents who noticed a change in general macrofungal abundance during the period of mushroom collection. In some cases, we collected reports on observed abundance changes of particular fungi. The collected data was used to create Macrofungi abundance decrease maps that recorded the main causes of these changes (Fig. 4). These maps were created on the basis of data collected in particular localities. Interpolations were made with the geometric interval method. Answers were classified as ‘anthropopressure’ when respondents mentioned human agents affecting the habitat in general without directly specifying official forest management. All maps were created using ArcMap 10.4.1.

## Results

### Habitats listed by locals to describe habitat preference of mushroom species

We found 98 habitat types mushroom collectors used to describe habitats of collected fungi (Table 1). Most habitats (65) may be regarded as mesic habitats (e.g. different forest types, such as coniferous forest, deciduous forest, mixed forest, pine forest, forest edges, openings), 28 as microhabitats (e.g. terrain elevations or hillocks, roadsides, tree stumps or fallen pine bark), and 4 as macro habitats (e.g. areas with or without forest vegetation).

Folk habitats referred to different characteristics of these habitats. The main dimensions were dominant species (e.g. *Pinus sylvestris* L*.*, *Populus tremula* L*.*), vegetation succession (clearcut, forest plantation, forest age, grass presence, deadwood presence, forest density, grass size), land-use type (forests, pastures, meadows, fields, fallows, wastelands, orchards, yards, stubbles, parks), vegetation structure (coniferous forest, deciduous forest, mixed forest, forest edge, forest cover and understory structure, hardwood forest), forest vegetation physiognomy (open forest, forest clearings, little exposure to sun, burned areas), geomorphology (terrain elevations, hills, hillocks, scarps, trenches, depressions, slopes, water edge), soils (sandy, fertile), hydrology (humid, dry, bogs), human and animal disturbances (roadsides, presence of tree stumps, presence of human-made structures, firebreaks, balks, boar rooting, manure presence, foxholes), and history of land use (forests on previously cultivated grounds).

### Observed habitat preference of mushroom species

Field data concerning local knowledge about collected fungi species habitat preferences acquired during the field research was compiled into collective habitat descriptions for 35 different fungal taxa, enabling the creation of quantitative graphs depicting the most important habitats determining particular fungi species occurrence (Fig. 2, Table 3).

The collected data allowed to group species according to seven macrohabitats (Table 2).

**Table 2:** Fungi habitat preferences according to the interviewees (Mazovia, Poland)

Grasslands	Forest clearcutting	Semi-open and light forest	Various habitats	Deciduous forest	Coniferous forest	Mixed forests
*Agaricus campestris*	*Armillaria mellea*	*Boletus edulis*	*Amanita vaginata*	*Armillaria mellea*	*Armillaria mellea*	*Amanita vaginata*
*Macrolepiota procera*	*Gyromitra esculenta*	*Boletus subtomentosus*	*Boletus subtomentosus*	*Boletus edulis*	*Boletus edulis*	*Boletus edulis*
*Marasmius oreades*	*Morchella* spp.	*Lactarius deliciosus*	*Cantharellus cibarius*	*Boletus reticulatus*	*Cantharellus cibarius*	*Boletus reticulatus*
	*Pleurotus ostreatus*	*Leccinum scabrum*	*Leccinum scabrum*	*Cantharellus cibarius*	*Cortinarius caperatus*	*Boletus subtomentosus*
		*Macrolepiota procera*	*Macrolepiota procera*	*Leccinum aurantiacum*	*Gyromitra esculenta*.	*Cantharellus cibarius*
		*Paxillus involutus*	*Paxillus involutus*	*Leccinellum pseudoscabrum*	*Gyroporus cyanescens*	*Cortinarius caperatus*
		*Russula* spp.	*Russula* spp.	*Leccinum scabrum*	*Hygrophorus hypothejus*	*Craterellus cornucopioides*
		*Suillus bovinus*	*Suillus bovinus*	*Leccinum scabrum*	*Imleria badia*	*Gyromitra esculenta*
				*Paxillus involutus*	*Lactarius deliciosus*	*Gyroporus cyanescens*
				*Russula* spp.	*Morchella* spp.	*Imleria badia*
					*Paxillus involutus*	*Lactarius deliciosus*
					*Russula* spp.	*Leccinum aurantiacum*
					*Sarcodon squamosus*	*Leccinum scabrum*
					*Suillus bovinus*	*Macrolepiota procera*
					*Suillus grevillei*	*Morchella* spp.
					*Suillus luteus*	*Paxillus involutus*
					*Suillus variegatus*	*Pleurotus ostreatus*
					*Tricholoma equestre*	*Russula* spp.
					*Tricholoma portentosum*	*Sarcodon squamosus*
						*Suillus bovinus*
						*Suillus variegatus*
						*Tricholoma equestre*
						*Tricholoma portentosum*

Figure 3 shows a clear correlation between open area habitats—such as fields, meadows, and roadsides—and particular species of fungi, such as the saprotrophic *Marasmius oreades* (Bolton) Fr*.*, *Agaricus campestris* L. or *Macrolepiota procera* (Scop.) Singer. *Leccinum* Gray spp. is closely correlated with birch and early successional habitats containing grasses. The top right part of the graph groups species correlated with dry, sandy, and disturbed soils (for example species from *Tricholoma* (Fr.) Staude, *Hygrophorus hypothejus* (Fr.) Fr. or *Gyromitra esculenta* (Pers.) Fr*.*). Habitats such as pine and moss are positively correlated, and they group species characteristic for pine forests, for example species from the *Suillus* genus. Species positively correlated with mixed forest habitats, birch forests, and a large number of various habitats are *Boletus subtomentosus* L*.*, *Paxillus involutus* (Batsch) Fr*.*, or species from the *Russula* Pers. genus.

### Abundance changes of fungi perceived by local mushroom collectors

Most respondents (53%) observed a decrease of macrofungi abundance during their lifetime (10–50 years). Among them, 12 respondents (2%) emphasized that the biggest drop in abundance of fruiting bodies occurred during the last two decades. The 13% of respondents who noticed fluctuations in abundance attributed them to natural changes related to annual differences in yearly rainfall and temperatures. Over a third (34%) of respondents did not notice any changes in fungal abundance. Only one person (0.14%) noticed a steady increase of macrofungi abundance.

Respondents mainly focused on general abundance of edible macrofungi species. The general view on mushroom abundance emerged from the assumption that the majority of fungal species react to the same biotic and abiotic stresses. According to the majority of reports, there has been a noticeable decrease in the abundance of all macrofungi in the whole Mazovia region (Fig. 4). This concerns especially the northern and western parts of the region, where over 70% of the respondents have noticed a decrease in macrofungal abundance. The main reason for abundance decrease listed by the informants is drought (*n* = 186, 27% of respondents). Reports of progressive drought negatively affecting fungal abundance were recorded in all 38 research localities. Other reasons were as follows: forest management (*n* = 30), climate change (*n* = 21), anthropopressure (*n* = 19), environmental pollution (*n* = 16), overgrowing habitats (*n* = 11), and wild boar activity (*n* = 5). Sixty respondents were not able to list the cause of declining macrofungal abundance.

The lowest percentage of decrease in fungal abundance (around 35%) was recorded in the eastern part of the Mazovia region. In this area, the most often listed determinant of mushroom abundance decrease was forest habitats becoming overgrown by understory vegetation. In the north-eastern part of Mazovia, where the decrease in abundance is highest, respondents have declared that ‘forest management’ is the main cause of this phenomenon. In localities situated close to the south-west of the capital city, anthropopressure has been determined as the main cause of edible fungi abundance decrease. Aside from overall information on macrofungal abundance, some of the respondents also noted a significant decrease in the abundance of particular fungi species. Altogether, 27 independent respondents reported a significant decrease of *Lactarius deliciosus* (L.) Gray abundance, 19—a decrease of *Boletus edulis* Bull. abundance, 18—in species from the *Tricholoma* (Fr.) Staude genus. Additionally, 8 respondents recorded a significant decrease of *Tricholoma equestre* (L.) P. Kumm. abundance, 18—a decrease of *Cantharellus cibarius* Fr. abundance, 12—a decrease of *Agaricus campestris* L. abundance, and 10—a decrease of *Suillus luteus* (L.) Roussel abundance. An increased abundance of one species, *Imleria badia* (Fr.) Vizzini, has also been noted, with its increase reported by 15 independent respondents (Table 3).

**Table 3:** Habitat preferences and abundance changes of selected fungal taxa.

Species	Habitat	Habitat (***n***)	Abundance changes			
			Increase (***n***)	Increase cause	Decrease (***n***)	Decrease cause
Macromycetes general	Table 1	Table 1	1	Imprecise	186	Drought
					60	Imprecise
					30	Forest management
					21	Climate changes
					19	Antropopression (general)
					16	Pollution
					11	Habitat overgrowing
					10	Mycelium/litter damage
					3	Boars
					3	Grazing abandonment
					3	Low night temperatures
					2	Incorrect collection
					2	Urbanization
					2	Increased pest activities
					1	Logging
					1	Unraked litter
					1	High night temperatures
Agaricus campestris s.l.	Meadow	104	0	None	12	Grazing abandonment
	Field	31				
	Cows/horses	31				
	Other	29				
Amanita vaginata	Various habitats	6	0	None	1	Forest management
	Mixed forest	5				
	Coniferous forest	3				
	Other	5				
Armillaria mellea s.l.	Tree stumps	100	0	None	2	Imprecise
	Old/tall forest	20				
	Clearcut area	18				
	Pine	17				
	Young/small forest	16				
	Deciduous forest	12				
	Dead wood	12				
	Humid ground	10				
	Oak	9				
	Alder	7				
	Other	61				
Boletus edulis s.l.	Oak	194	0	None	9	Imprecise
	Pine	158			6	Drought
	Mixed forest	102			2	Pollution
	Birch	90			1	Antropopression (general)
	Coniferous forest	32			1	Forest management
	Forest edge	30				
	Deciduous forest	26				
	Old/tall forest	26				
	Other	189				
Boletus reticulatus	Oak	13	0	None	0	None
	Sandy ground	5				
	Birch	3				
	Mixed forest	2				
	Other	4				
Boletus subtomentosus s.l.	Mixed forest	27	0	None	1	Imprecise
	Pine	15				
	Various habitats	14				
	Birch	9				
	Moss	5				
	Forest edge	4				
	Grasses	4				
	Other	42				
Cantharellus cibarius s.l.	Mixed forest	106	0	None	13	Imprecise
	Pine	99			5	Drought
	Birch	64				
	Oak	43				
	Moss	37				
	Sandy ground	35				
	Coniferous forest	32				
	Deciduous forest	20				
	Various habitats	20				
	Other	127				
Cortinarius caperatus	Pine	32	0	None	2	Drought
	Moss	20			1	Forest management
	Mixed forest	16			1	Imprecise
	Coniferous needles	16				
	Old/tall forest	12				
	Birch	8				
	Sunny areas	8				
	Clear/transparent forest	8				
	Other	52				
Craterellus cornucopoides	Pine	4	0	None	2	Imprecise
	Oak	4				
	Mixed forest	2				
Gyromitra esculenta	Pine	10	0	None	0	None
	Forest plantation	8				
	Young/small forest	5				
	Clearcut area	4				
	Sandy ground	3				
	Mixed forest	2				
	Firebreaks	2				
	Other	6				
Gyroporus cyanescens	Sandy ground	21	0	None	2	Imprecise
	Pine	7				
	Roadside	6				
	Oak	4				
	Yard	4				
	Forest edge	3				
	Young/small forest	3				
	Juniperus spp.	2				
	Moss	2				
	Other	2				
Hygrophorus hypothejus	Pine	9	0	None	0	None
	Coniferous forest	3				
	Young/small forest	3				
	Moss	2				
	‘Man's forest’	2				
	Other	6				
Imleria badia	Pine	200	15	Imprecise	2	Imprecise
	Mixed forest	82				
	Moss	61				
	Coniferous forest	51				
	Various habitats	20				
	Other	163				
Lactarius deliciosus s.l.	Grasses	29	0	None	16	Drought
	Pine	24			8	Imprecise
	Forest edge	15			2	Forest management
	Meadow	15			1	Pollution
	Mixed forest	14				
	Trenches/depressions	14				
	Coniferous forest	12				
	Spruce	10				
	Oak	7				
	Moss	7				
	Humid ground	7				
	Other	50				
Leccinum aurantiacum s.l.	Birch	175	0	None	3	Drought
	Aspen	69			1	Pollution
	Mixed forest	33				
	Deciduous forest	26				
	Alder	15				
	Other	121				
Leccinum pseudoscabrum	Hornbeam	7	0		3	Imprecise
	Old/tall forest	4				
	Humid ground	2				
	Bog	2				
	Birch	2				
	Other	3				
Leccinum scabrum	Birch	195	0	None	1	Imprecise
	Mixed forest	38			1	Drought
	Grass	16				
	Pine	14				
	Various habitats	14				
	Other	97				
Leccinum spp.	Birch	137	0	None	2	Drought
	Mixed forest	29				
	Oak	14				
	Pine	11				
	Other	99				
Macrolepiota procera	Meadow	78	0	None	1	Imprecise
	Forest edge	50				
	Field	36				
	Mixed forest	33				
	Various habitats	19				
	Open area	16				
	Grasses	13				
	Roadsides	12				
	Pine	11				
	Fallow	10				
	Other	91				
Marasmius oreades	Roadside	16	0	None	2	Grazing abandonment
	Cows/horses	13				
	Yard	7				
	Meadow	4				
	Trenches/depressions	2				
	Other	8				
Morchella esculenta s.l.	Pine	5	0	None	2	Habitat overgrowing
	Clear-cut area	3				
	Fallen bark	3				
	Oak	2				
	Mixed forest	2				
	Moss	2				
	Other	4				
Paxillus involutus s.l.	Mixed forest	20	0	None	4	Drought
	Various habitat	12			1	imprecise
	Pine	7				
	Deciduous forest	4				
	Other	15				
Pleurotus ostreatus	Mixed forest	2	0	None	0	None
	Dead wood	2				
	Clear-cut area	2				
	Other	4				
Russula aeruginea s.l.	Mixed forest	13	0	None	2	Imprecise
	Various habitats	9			1	Drought
	Birch	7				
	Coniferous forest	4				
	Pine	3				
	Forest edge	2				
	Grass	2				
	Other	7				
Russula integra s.l.	Birch	3	0	None	0	None
	Coniferous forest	2				
	Various habitats	2				
	Mixed forest	2				
	Other	2				
Russula virescens	Birch	4	0	None	2	Imprecise
	Various habitats	2				
	Other	4				
Russula spp.	Mixed forest	22	0	None	2	Drought
	Various habitats	11			1	Antropopression (general)
	Pine	4			1	Imprecise
	Birch	4				
	Coniferous forest	3				
	Deciduous forest	3				
	Oak	3				
	Grasses	3				
	Other	10				
Sarcodon squamosus	Pine	14	0	None	2	Drought
	Coniferous forest	8			2	Forest management
	Old/tall forest	8			1	Imprecise
	Elevations/hillocs	5				
	Mixed forest	3				
	Other	3				
Suillus bovinus	Pine	15	0	None	1	Drought
	Various habitats	7			1	Imprecise
	Mixed forest	4				
	Young/small forest	4				
	Forest edge	3				
	Elevations/hillocks	3				
	Grasses	3				
	Coniferous forest	2				
	Moss	2				
	Blackberries	2				
	Humid ground	2				
	Boar rooting	2				
	Other	6				
Suillus grevillei	Larch	12	0	None	0	None
	Other	1				
Suillus luteus s.l.	Pine	239	0	None	5	Imprecise
	Young/small forest	204			3	Drought
	Coniferous forest	78			2	Antropopression (general)
	Grasses	20				
	Thickets	19				
	Other	112				
Suillus variegatus	Pine	19	0	None	1	Imprecise
	Humid ground	7				
	Young/small forest	7				
	Mixed forest	5				
	Coniferous forest	4				
	Moss	4				
	Deciduous forest	2				
	Juniper	2				
	Elevations/hillocks	2				
	Grasses	2				
	Blueberries	2				
	Clear/transparent forest	2				
	Trenches/depressions	2				
	Other	10				
Tricholoma equestre	Sandy ground	82	0	None	5	Imprecise
	Pine	78			2	Antropopression (general)
	Elevations/hillocks	47			1	Habitat overgrowing
	Coniferous forest	21				
	Young/small forest	20				
	Moss	19				
	Mixed forest	12				
	Other	61				
Tricholoma portentosum	Sandy ground	77	0	None	1	Antropopression (general)
	Pine	62			1	Drought
	Elevations/hillocks	43			2	Imprecise
	Coniferous forest	23				
	Moss	18				
	Young/small forest	18				
	Mixed forest	10				
	Roadside	10				
	Other	54				
Tricholoma spp.	Pine	124	0	None	10	Imprecise
	Sandy ground	118			8	Drought
	Elevations/hillocks	54				
	Coniferous forest	33				
	Mixed forest	28				
	Young/small forest	28				
	Moss	24				
	Other	79				

## Discussion

### Habitats listed to describe habitat preference

While describing fungi habitats, mushroom collectors mentioned 98 habitat types, of which most were meso- and microhabitats. Local ethnoecological knowledge on fungi was formed at a finer spatial scale than knowledge concerning plant ethnoecology documented in previous research [10, 15, 16].

Respondents usually described tree species only to the genus level. The respondents gave detailed descriptions of forest communities relatively rarely. However, they mentioned some very specific fungal habitats like hillocks, firebrakes, self-sown forests, specific litter layer composition, or relevant tree species, as these features enable them to specify the landscape in which they usually look for certain species of fungi, implementing high complexity of folk knowledge related to fungal ecology. On the other hand, in folk ecology descriptions, we can find recurring habitat characteristics that are still not scientifically evaluated in depth in relation to fungi occurrence. These include exposure to sun (mentioned particularly often), the shape of the terrain, or litter thickness. Such indicators were very often perceived as crucial during the description of particular fungi species habitats. This information may provide new guidelines that could determine the direction of further studies on ecology of local fungi.

Respondents determined habitats using diverse dimensions (see section “Habitats listed by locals to describe habitat preference of mushroom species”). Studies conducted with other local communities show that these dimensions are shaped by different environments that make them characteristic for certain local groups [15]. When comparing dimensions used to determine fungal habitats with dimensions used by different communities, we can notice some similarities. The most important dimensions recorded in the present study, such as dominant species, land-use type, or vegetation structure, are characteristic for local communities living in the Carpathians and are less important to people living in Western Canada or Mongolia [16]. This suggests similarities in habitat perception between Central European communities that are worthy of further investigation.

Respondents described coniferous forests as richer in fungi species than decidous forests. However, this is not reflected in scientific studies [39]. This result might be related to the composition of local forests. These forests are dominated by pine, which often creates monocultures and is included in mixed woodlands [31]. Therefore, coniferous forests are visited most often, which makes respondents more familiar with the composition of coniferous forest fungi.

### Observed habitat preference of mushroom species

Data provided by scientific publications seldom displays information which habitat characteristics have the biggest importance for the development of a particular species. The large number of interviewees allows us to define the significance of particular habitat indicators based on the percentage of the most often mentioned characteristics.

By analysing the most frequently mentioned fungal habitats, we were able to create collective ethnoecological descriptions with characteristics comparable to scientific knowledge. Comparison of local folk habitat descriptions with the available scientific knowledge allowed us to select those observations which are present in scientific literature or need further investigation (Table 4).

**Table 4:** Evaluation of reports of Polish mushroom collectors by present scientific mycological knowledge

Reports correspond with scientific literature	Mentioned as possible in literature but not yet tested	Not mentioned in literature and not yet tested
The importance of grazing areas and animal manure for the abundance of saprotrophic fungi such as *Agaricus campestris*, *Marasmius oreades*, and *Macrolepiota procera* [40–43]	The xerophillic character of *Amanita vaginata*. Unconfirmed for *A. vaginata* but confirmed for some species from the *Vaginatae* section [44]	Higher abundance of *Hygrophorus hypothejus*’s fruiting bodies in pine forests growing on former arable land than in ancient forest locations
*Leccinum scabrum*’s preference for sylvopastoral habitats [39]	High amplitudes of litter temperature as a stimulator of the production of fruiting bodies	*Suillus bovinus*, *Tricholoma equestre* and *Tricholoma portentosum* abundance is higher on uneven ground surface
*Armillaria* spp.’s preference towards living on young pine trees – the fungus’ ability to produce fruiting bodies decreases with the age of the infected pine tree [45, 46]	Low canopy density and exposure of litter to sun stimulating the fruiting of *Cortinarius caperatus* [47]	Litter density as one of the main factors determining particular *Suillus* species fructification
*Hygrophorus hypothejus’*, *Suillus bovinus*’, and *Suillus luteus’* preference towards young pine forest stands [48–52]	Higher presence of *Pleurotus ostreatus* in cutting and managed areas [53, 54]	Boar rooting as a stimulator of the production of *Suillus bovinus* fruiting bodies
*Boletus edulis’*, *Cortinarius caperatus’*, *Sarcodon squamosus’* preference towards old forest stands [55–58]	The positive effect of forest age on the abundance of production of fungal fruiting bodies [59]	The declining abundance of saprotrophic fungi in analysed areas as related to grazing abandonment and the use of synthetic fertilizers
*Armillaria mellea*’s need for relatively higher moisture than other wood-decaying basidiomycetes [60]	Influence of moss on the fungal fruiting process (e.g. its protective effect, increasing soil nitrogen and phosphorus content and being the source of saprobiotic nutrition) [61–65]	
Higher abundance *of Lactarius deliciosus* fruiting bodies in trenches and small depressions – the appropriate slope and elevation are significant predictors of Lactarius deliciosus [66, 67]		
*Lactarius deliciosus’* complex requirement for high moisture in conjunction with access to strong sunlight [47, 66, 68–70]		
*Suillus bovinus’* preference for relatively higher moisture than other macrofungi [50, 71, 72]		
Moss presence as one of the parameters potentially determining the habitat of *Cantharellus cibarius*, *Cortinarius caperatus* and *Suillus bovinus* [61, 63, 73, 74]		
*Suillus bovinus* and *Suillus luteus* fruiting bodies’ occurrence on thin litter layer [48, 51, 75]		
*Suillus variegatus* fruiting bodies’ occurrence on thick litter layer [76]		
Broken or ploughed forest cover inducing the production of *Gyromitra esculenta* and *Morchella* spp. ascocarps [77–80]]		
Higher abundance of *Boletus edulis*, *Boletus subtomentosus* and *Russulaceae* fruiting bodies in lighter forest areas such as forest edges [81–83]		

The following folk observations correspond to already published scientific reports:

1. The importance of grazing areas and animal manure for the abundance of saprotrophic fungi such as *Agaricus campestris* L., *Marasmius oreades* (Bolton) Fr. and *Macrolepiota procera* (Scop.) Singer [40–43].
2. *Leccinum scabrum’s* (Bull.) Gray preference for sylvopastoral habitats [39];
3. *Armillaria* (Fr.) Staude spp.’s preference towards living young pine trees—fungus’ ability to produce fruiting bodies decreases with the age of the infected pine tree [45, 46];
4. *Hygrophorus hypothejus’* (Fr.) Fr*.*, *Suillus bovinus’* (L.) Roussel, and *Suillus luteus’* (L.) Roussel preference towards young pine forest stands [48–52];
5. *Boletus edulis’* Bull., *Cortinarius caperatus’* (Pers.) Fr*.*, *Sarcodon squamosus’* (Schaeff.) Quél. preference towards old forest stands [55–58];
6. *Armillaria mellea’s* (Vahl) P. Kumm*.* s.l. need for relatively higher moisture than other wood-decaying basidiomycetes [60];
7. Higher abundance of *Lactarius deliciosus* (L.) Gray s.l. fruiting bodies in trenches and small depressions—the appropriate slope and elevation are significant predictors of *Lactarius deliciosus* (L.) Gray s.l. [66, 67];
8. *Lactarius deliciosus’* (L.) Gray complex requirement for high moisture in conjunction with access to strong sunlight [47, 66, 68–70];
9. *Suillus bovinus’* (L.) Roussel preference for relatively higher moisture than other macrofungi [50, 71, 72];
10. Moss presence as one of the parameters potentially determining the habitat of *Cantharellus cibarius* Fr*.*, *Cortinarius caperatus* (Pers.) Fr. and *Suillus bovinus* (L.) Roussel [61, 63, 73, 74];
11. *Suillus bovinus* (L.) Roussel and *Suillus luteus* (L.) Roussel fruiting bodies’ occurrence on thin litter layer [48, 51, 75];
12. *Suillus variegatus* (Sw.) Richon & Roze fruiting bodies’ occurrence on thick litter layer [76];
13. Broken or ploughed forest cover inducing the production of *Gyromitra esculenta* (Pers.) Fr. and *Morchella* Dill. ex Pers. spp. ascocarps [77–80];
14. Higher abundance of *Boletus edulis* Bull., *Boletus subtomentosus* L. and *Russulaceae* Lfruiting bodies in lighter forest areas such as forest edges [81–83].

Some phenomena observed by the informants have not yet been researched or tested by science, e.g.:

1. Higher abundance of *Hygrophorus hypothejus’s* (Fr.) Fr. fruiting bodies in pine forests growing on former arable land than those in ancient forest locations;
2. *Suillus bovinus* (L.) Roussel, *Tricholoma equestre* (L.) P. Kumm*.* and *Tricholoma portentosum* (Fr.) Quél. abundance is higher on uneven ground surface;
3. Litter density as one of the main factors determining particular *Suillus* species fructification;
4. Boar rooting as a stimulator of the production of *Suillus bovinus* (L.) Roussel fruiting bodies;
5. The declining abundance of saprotrophic fungi in analysed areas as related to grazing abandonment and the use of synthetic fertilizers.

Some phenomena mentioned by informants are known to many mycologists but have no scientific confirmation or were only suggested by some authors:

1. The xerophillic character of *Amanita vaginata* (Bull.) Lam*.* Unconfirmed for *A. vaginata*, but confirmed for some species from the *Vaginatae* section [44];
2. High amplitudes of litter temperature as a stimulator of the production of fruiting bodies;
3. Low canopy density and exposure of litter to sun stimulating the fruiting of *Cortinarius caperatus* (Pers.) Fr.—unconfirmed, but recent studies show its lower abundance in relatively high moisture conditions [47], which might be connected with low sun exposure;
4. Higher presence of *Pleurotus ostreatus* (Jacq.) P. Kumm*.* in cutting and managed areas; unconfirmed but suggested by a few authors (dead and damaged wood presence, wood inoculation) (e.g. [53, 54]);
5. The positive effect of forest age on the abundance of production of fungal fruiting bodies; mainly unexplored with one publication contradicting it [59];
6. Influence of moss on fungal fruiting process (e.g. protective effect, increasing soil nitrogen and phosphorus content and source of saprobiotic nutrition); mostly unexplored but suggested by [61–65].

### Perceived abundance change of mushrooms

Mushroom collectors had the general perception that the decrease of mushroom abundance is the general trend in the areas they visit to collect mushrooms. The steady decrease of macrofungal abundance in Europe was already noticed in the 1970s [84–86]. At the beginning of the 1990s, scientists started to talk about the Mass Extinction of European Fungi [87, 88]. However, this tendency was formulated only on the basis of single reports, without presentation of any statistical figures [89].

The extensive research on the decline in the abundance of macrofungi was initiated at the end of the 1980s by the Dutch scientist, Eef Arnolds. The declining abundance of saprotrophic species occurring in the grasslands has been recorded mostly in connection to the newly implemented agricultural practices and use of artificial fertilizers [89]. A similar correlation has also been noticed by people living in Mazovia. When reporting on the abundance decrease of the field mushroom (*Agaricus campestris* L.) (12 persons), respondents stated grazing abandonment, changes in agricultural practices, and application of artificial fertilizers as the main causes of their decline. Arnolds [89] noticed a significant abundance decrease of 55 out of 126 analysed fungal species. It was mainly related to species characteristic of coniferous forests, which is the dominating type of forest in Mazovia (64%). Air and soil pollution were taken to be the main cause of the decreasing abundance of macrofungi [89–91]. Arnolds based his research on long-term field observations preceding data analysis (1912–1954 and 1973–1982 as well as data collected during two decades of individual research preceding its publication). The results of the analysis showed a drop in the number of macrofungi species occurring in the Netherlands from 37 to 12 per 1000 m^2^. Similarly, as in case of studies contacted in Mazovia, Arnolds [89] observed that species which suffered the most significant decrease belonged to the *Lactarius*, *Cantharellus*, *Boletus*, *Tricholoma*, and *Suillus* genus. According to his studies, the biggest abundance decrease is observed among ectomycorrhizal fungi species—a group to which the majority of species mentioned in present work belong to. However, Arnolds did not take the gradual changes occurring in soil water regimes into consideration. According to recent studies on soil water content changes, in the last few decades we have been dealing with a gradual decrease of soil water content in Poland [91–93]. Respondents, too, listed it as one of the main reasons for the decrease in fungal abundance in Mazovian forests (Fig. 4).

Current studies also confirm Arnolds’ reports on the visible decrease of macrofungi abundance. Research from Norway [94] confirms the significantly negative influence of nitrogen fertilization on the occurrence of fungal fruiting bodies. However, the same research also shows a high influence of drought on the decrease in the production of fruiting bodies. Studies conducted in northern Spain proved that partial rain exclusion (− 30%) lowered the production of fungal fruiting bodies by 60% [95]. De Aragón et al. [96] noticed that the right balance between accumulated monthly mean precipitation and evapotranspiration was of the greatest importance for macrofungi occurrence.

It was established that the main indicators of basidiomycetes’ fruiting bodies presence are soil moisture and its temperature back in the mid-20th century [97]. Certain levels of these indicators have to occur simultaneously for a period of time relevant to the particular species. While all different species depend on different ranges of temperature, all species rely on an increased level of soil moisture. Dahlberg [98] showed that similar weather conditions can determine the production of 55–88% fruiting bodies of basidiomycetes species (after [94]).

The impact of climate change on fungi is scientifically indisputable. Gange [99] conducted 56-year-long research on the period of macrofungal fructification. Data collected on 315 different species shows a tendency for the average first date of fructification to come earlier in the year as time goes on, while the average last fruiting date now occurs significantly later. In his studies on climate change, Schär et al. [100] focused not on the gradual rise of temperatures, but on increasing temperature variability in Central Europe. According to his observations, one of the main results of this phenomenon is summer droughts such as the one which occurred in Poland in 2003 [101]. The progressive drought observed by the respondents, with its impact on changes in local mycobiota, might be related to scientifically observed changes in climate.

It has been recognized that the act of mushroom picking has no significant impact on macrofungal fruiting body abundance [102]. Mycorrhiza compression, on the other hand, can have a large impact on the occurrence of fruiting bodies. During present research, 10 independent respondents noticed a relationship between lower numbers of mushrooms and the introduction of heavy machinery to forest management. According to their reports, the abundance of fungal fruiting bodies decreased after band-saw operators were replaced with devices equipped with felling heads. The highly negative impact of the pressure of heavy machinery on forest litter layer has been confirmed by Arnolds [91] and Frey [103]. The correlation between heavy machinery use and mushroom abundance decrease is so significant that it is visible to a respondent’s naked eye. Therefore, it is important to conduct further studies on the scale of this problem and to search for a new solution to be implemented in forest management. The decrease in fungal abundance could be also related to disturbances in the environmental nitrogen cycle as a result of artificial manure use, as confirmed by Vitousek [104].

The increased abundance of *Imleria badia* (Fr.) Vizzini, as observed by 15 respondents, can be explained by the Bay Bolete’s high capacity to adapt to habitats with acidic soils [105]. This type of soil dominates in pine forests—the main forest type in Mazovia. The research conducted in European countries by Rosinger el al [106]. shows that species such as *Xerocomus badius* (Fr.) E.-J. Gilbert (currently *Imleria badia* (Fr.) Vizzini), *Scleroderma citrinum* Pers. and *Paxillus involutus* (Batsch) Fr. usually occur in areas that combine high annual temperature and low annual rainfall. This may also explain the higher *Imleria badia* occurrence. Furthermore, Clemmensen [107], Morgado [108], and Fernandez [109] classify the Bay Bolete to the group of long-distance exploration fungi. In other words, this species is able to create long rhizomorphs that enable efficient habitat penetration. Aside from improving its ability to explore, long rhizomorphs also improve water transportation and accumulation [110].

## Conclusions

The interviewed Polish mushroom collectors had a deep understanding of fungal habitats. They used different scales of habitats to describe the habitat preferences of various fungi species. The high number of 98 fungal habitats listed by the respondents confirms the highly mycophillic character of people living in the studied area [34]. We found that some phenomena which have not yet been studied or tested by science were observed by multiple informants. Locals had the unanimous perception that fungal abundance is decreasing, and they identified drought as the key driver of the change.

We conclude that local ecological knowledge of lay mushroom collectors could offer new stimuli for scientific research and contribute to citizen-based monitoring of macrofungi.

Our large area study on fungal ethnoecology has a preliminary character and aims to encourage further research on this topic in other regions inhabited by mycophillic societies.

## Data Availability

Voucher specimens for species were deposited in the herbarium of Warsaw University (WAW).

## References

[1] Conklin HC (1957). Hanunoo Agriculture. FAO Forestry Development Paper No. 12.

[2] Anderson EN, Anderson EN, Pearsall DM, Hunn ES, Turner NJ (2011). Ethnobiology: overview of a growing field. Ethnobiology.

[3] Ingold T. The perception of the environment: essays on livelihood, dwelling and skill. London: Routledge.; 2002.

[4] Berkes F (1993). Traditional ecological knowledge in perspective. Traditional ecological knowledge: concepts and cases (Vol. 1).

[5] Berlin EA, Berlin B. Medical ethnobiology of the Highland Maya of Chiapas, Mexico: the gastrointestinal diseases. 2015;10(2):271–3. 10.1002/(sici)1520-6300(1998)10:2<271::aid-ajhb14>3.0.co;2-5. 10.1002/(SICI)1520-6300(1998)10:2<271::AID-AJHB14>3.0.CO;2-5 28561443

[6] Nazarea VD, editor. Ethnoecology: situated knowledge/located lives.Tucson: University of Arizona Press; 1999.

[7] Yamin-Pasternak S, Pasternak I. Ethnomycology. Int Encycl Anthropol. 2018:1–2. 10.1002/9781118924396.wbiea2088.

[8] Huntington HP. Using traditional ecological knowledge in science: methods and applications. Ecol Appl. 2000;10(5):1270–4. 10.1890/1051-0761(2000)010[1270:UTEKIS]2.0.CO;2.

[9] Moller H, Berkes F, Lyver POB, Kislalioglu M. Combining science and traditional ecological knowledge: monitoring populations for co-management. Ecol Soc. 2004;9(3). 10.5751/es-00675-090302.

[10] Molnár Z, Kis J, Vadász C, Papp L, Sándor I, Béres S, et al. Common and conflicting objectives and practices of herders and conservation managers: the need for a conservation herder. Ecosystem Health Sustainability. 2016;2(4):e01215.

[11] Gantuya B, Avar Á, Babai D, Molnár Á, Molnár Z (2019). “A herder’s duty is to think”: landscape partitioning and folk habitats of Mongolian herders in a mountain forest steppe (Khuvsugul-Murun region). J Ethnobiol Ethnomed.

[12] Bürgi M, Bieling C, Von Hackwitz K, Kizos T, Lieskovský J, Martín MG, Printsmann A (2017). Processes and driving forces in changing cultural landscapes across Europe. Landscape Ecol.

[13] Kelemen E, Nguyen G, Gomiero T, Kovács E, Choisis JP, Choisis N, Paoletti MG, Podmaniczky L, Ryschawy J, Sarthou JP, Herzog F, Dennis P, Balázs K, Herzog F (2013). Farmers’ perceptions of biodiversity: lessons from a discourse-based deliberative valuation study. Land use Policy.

[14] Nakashima D, KrupniEk I, Rubis JT, editors. Indigenous knowledge for climate change assessment and adaptation.Cambridge: Cambridge University Press; 2018.

[15] Ujházy N, Molnár Z, Bede-Fazekas Á, Biró M. Do farmers and conservationists perceive landscape changes differently? Ecol Soc. 2020;25(3):12.

[16] Ahnström J, Höckert J, Bergeå HL, Francis CA, Skelton P, Hallgren L (2009). Farmers and nature conservation: What is known about attitudes, context factors and actions affecting conservation?. Renew Agri Food Syst.

[17] Gadgil M, Seshagiri Rao PR, Utkarsh G, Pramod P, Chhatre A (2000). New meanings for old knowledge: the people’s biodiversity registers program. Ecol Appl.

[18] Volpato G, Rossi D, Dentoni D (2013). A reward for patience and suffering: ethnomycology and commodification of desert truffles among Sahrawi refugees and nomads of Western Sahara. Econ Bot.

[19] Trappe JM, Claridge AW, Claridge DL, Liddle L (2008). Desert truffles of the Australian outback: ecology, ethnomycology, and taxonomy. Econ Bot.

[20] Lampman AM (2005). Tzeltal ethnomycology: naming, classification and use of mushrooms in the highlands of Chiapas, Mexico.

[21] Davies N (2012). Boże igrzysko. Historia Polski.

[22] Pałucki W (1973). Mazowsze w drugiej połowie XVI wieku.

[23] Główny Urząd Statystyczny. Bank Danych Lokalnych. 2018. Available at: https://bdl.stat.gov.pl/BDL/dane/podgrup/temat [cited 18.06.2018].

[24] Kondracki J (2000). Geografia regionalna Polski. Wydawn.

[25] Lorenc H (2005). Atlas klimatu Polski.

[26] European Environment Agency. Corine Land Cover 2012 Seamless Vector Data. 2017. Available at: https://www.eea.europa.eu/data-and-maps/data/clc-2012-vector [cited 18.06.2018].

[27] Braun K (1999). Oblicze etnograficzne współczesnego województwa mazowieckiego. Rocznik Mazowiecki.

[28] Martin GJ (2010). Ethnobotany: a methods manual.

[29] Kotowski MA, Pietras M, Łuczaj Ł (2019). Extreme levels of mycophilia documented in Mazovia, a region of Poland. J Ethnobiol Ethnomed.

[30] Albuquerque UP, Ramos MA, de Lucena RFP, Alencar NL (2014). Methods and techniques used to collect ethnobiological data. In Methods and techniques in Ethnobiology and Ethnoecology.

[31] Yamin-Pasternak S (2011). Ethnomycology: fungi and mushrooms in cultural entanglements. Ethnobiology.

[32] Lê S, Josse J, Husson F. FactoMineR: an R package for multivariate analysis. J Stat Softw. 2008;25(1, 1):–18. 10.18637/jss.v025.i01.

[33] Gumińska B, Wojewoda W (1985). Grzyby i ich oznaczanie.

[34] Wojewoda W (2003). Checklist of Polish Larger Basidiomycetes.

[35] Arora D (1986). Mushrooms demystified.

[36] Crisan EV, Sands A, Chang ST, Hayes WA. The biology and cultivation of edible mushrooms. Nutri Value N Y Acad. 1978:251–93. 10.1016/c2013-0-10484-9.

[37] Gramss G. The universe of basidiomycetous ground fungi. In: Current research, technology and education topics in applied microbiology and microbial biotechnology. Badajoz: Formatex; 2010. p. 218–29.

[38] Ingold CT, Hudson HJ (1993). Ecology of saprotrophic fungi. The Biology of Fungi.

[39] Abate D (1999). Agaricus campestris in upland Ethiopia. Mycologist.

[40] Shaw CG, Roth LF (1978). Control of Armillaria root rot in managed coniferous forests 1: a literature review. Eur J Forest Pathol.

[41] Termorshuizen AJ (1991). Succession of mycorrhizal fungi in stands of Pinus sylvestris in the Netherlands. J Veg Sci.

[42] Dahlberg A, Stenlid JAN (1994). Size, distribution and biomass of genets in populations of Suillus bovinus (L.: Fr.) Roussel revealed by somatic incompatibility. New Phytol.

[43] Kubiak J (2007). Wpływ różnych szczepionek mikoryzowych na wzrost sosny i liczbę pączków. Inżynieria Rolnicza.

[44] Piškur B, Bajc M, Robek R, Humar M, Sinjur I, Kadunc A, Jurc D (2011). Influence of Pleurotus ostreatus inoculation on wood degradation and fungal colonization. Bioresource Technol.

[45] Babai D, Molnár Z. Multidimensionality and scale in a landscape ethnoecological partitioning of a mountainous landscape (Gyimes, Eastern Carpathians, Romania). J Ethnobiol Ethnomed. 2013;9(1):1–21. 10.1186/1746-4269-9-11. 10.1186/1746-4269-9-11 PMC3610200 23388111

[46] Kałucka I (2009). Macrofungi in the secondary succession on the abandoned farmland near the Białowieża old-growth forest. Monog Botan.

[47] Hurst J, Rutherford L (1996). A gourmet’s guide to mushrooms and truffles.

[48] Kałucka IL, Jagodziński AM (2016). Successional traits of ectomycorrhizal fungi in forest reclamation after surface mining and agricultural disturbances: a review. Dendrobiology.

[49] Durall DM, Gamiet S, Simard SW, Kudrna L, Sakakibara SM (2006). Effects of clearcut logging and tree species composition on the diversity and community composition of epigeous fruit bodies formed by ectomycorrhizal fungi. Botany.

[50] Stankeviciene D, Kasparavicius J (2007). Studies on ectomycorrhizal basidiomycete in pine forest on the Lithuania-Poland transboundary region. Acta Mycol.

[51] Martínez-Peña F, Ágreda T, Águeda B, Ortega-Martínez P, Fernández-Toirán LM (2012). Edible sporocarp production by age class in a Scots pine stand in Northern Spain. Mycorrhiza.

[52] Eilertsen L. Presence of Hydnellum species on pine heaths. Umeå: Umeå University; 2014.

[53] Łuczaj Ł, Sadowska B (1997). Edge effect in different groups of organisms: vascular plant, bryophyte and fungi species richness across a forest-grassland border. Folia Geobotan Phytotaxonom.

[54] Lavoie M, Paré D, Bergeron Y (2006). Relationships between microsite type and the growth and nutrition of young black spruce on post-disturbed lowland black spruce sites in eastern Canada. Can J Forest Res.

[55] Boddy L (2000). Interspecific combative interactions between wood-decaying basidiomycetes. FEMS Microbiol Ecol.

[56] Bonet JA, De-Miguel S, de Aragón JM, Pukkala T, Palahí M (2012). Immediate effect of thinning on the yield of Lactarius group deliciosus in Pinuspinaster forests in Northeastern Spain. Forest Ecol Manage.

[57] Kucuker MD, Baskent EZ (2019). Modeling the productivity of commercial Lactarius mushrooms: a case study in the Kizilcasu planning unit, Turkey. Nat Res Model.

[58] Zhu JJ, Li FQ, Xu ML, Kang HZ, Wu XY (2008). The role of ectomycorrhizal fungi in alleviating pine decline in semiarid sandy soil of northern China: an experimental approach. Ann Forest Sci.

[59] Johnson LM, Hunn ES. Landscape ethnoecology: concepts of biotic and physical space. New York: Berghahn Books; 2010. 10.1080/00207233.2012.688466.

[60] Hobson G. Traditional knowledge is science. Northern Perspect. 1992;20(1):2.

[61] Hintikka V (1988). On the macromycete flora in oligotrophic pine forests of different ages in South Finland. Acta Bot Fenn.

[62] Koide RT, Malcolm GM (2009). N concentration controls decomposition rates of different strains of ectomycorrhizal fungi. Fungal Ecol.

[63] Aučina A, Rudawska M, Leski T, Skridaila A, Riepšas E, Iwanski M (2007). Growth and mycorrhizal community structure of Pinus sylvestris seedlings following the addition of forest litter. Appl. Environ. Microbiol..

[64] Bödeker IT, Clemmensen KE, de Boer W, Martin F, Olson Å, Lindahl BD (2014). Ectomycorrhizal Cortinarius species participate in enzymatic oxidation of humus in northern forest ecosystems. New Phytologist.

[65] Reid, D. A. Changes in the British macromycete flora. In The Changing Flora and Fauna of Britain (cd. D. L. Hawksworth), pp. 1974;79-85. Londyn, Nowy Jork.

[66] Pinna S, Gévry MF, Côté M, Sirois L (2010). Factors influencing fructification phenology of edible mushrooms in a boreal mixed forest of Eastern Canada. Forest Ecol Manage.

[67] Liu B, Bonet JA, Fischer CR, de Aragón JM, Bassie L, Colinas C (2016). Lactarius deliciosus Fr. soil extraradical mycelium correlates with stand fruitbody productivity and is increased by forest thinning. Forest Ecol Manage.

[68] Castaño C, Alday JG, Parladé J, Pera J, de Aragón JM, Bonet JA (2017). Seasonal dynamics of the ectomycorrhizal fungus Lactarius vinosus are altered by changes in soil moisture and temperature. Soil Biol Biochem.

[69] Sotek Z, Stasińska M (2010). Różnorodność macromycetes na tle przemian roślinności na torfowisku atlantyckim" Stramniczka". Woda-Środowisko-Obszary Wiejskie.

[70] Carleton TJ, Read DJ (1991). Ectomycorrhizas and nutrient transfer in conifer–feather moss ecosystems. Can J Bot.

[71] Pilz, D., Norvell, L., Danell, E., & Molina, R. Ecology and management of commercially harvested chanterelle mushrooms. Gen. Tech. Rep. PNW-GTR-576. Portland, OR: US Department of Agriculture, Forest Service, Pacific Northwest Research Station. 2003;83 p, 576. DOI: 10.2737/PNW-GTR-576

[72] Kauserud H, Mathiesen C, Ohlson M (2008). High diversity of fungi associated with living parts of boreal forest bryophytes. Botany.

[73] Rochon C, Paré D, Pélardy N, Khasa DP, Fortin JA (2001). Ecology and productivity of Cantharellus cibarius var. roseocanus in two eastern Canadian jack pine stands. Botany.

[74] Veijalainen H. Effect of forestry on the yields of wild berries and edible fungi. Ecol Bull. 1976:63–5.

[75] Jalkanen R, Jalkanen E (1978). Studies on the effects of soil surface treatments on crop of false morel (Gyromitra esculenta) in spruce forests. Karstenia.

[76] Omura K. Science against modern science: the socio-political construction of otherness in Inuit TEK (traditional ecological knowledge). Senri Ethnol Stud. 2005;67, 323-344.

[77] Ohenoja E (1988). Effect of forest management procedures on fungal fruit body production in Finland. Acta Bot Fenn.

[78] Goldway M, Rachel AMIR, Goldberg D, Hadar Y, Levanon D (2000). Morchella conica exhibiting a long fruiting season. Mycological Research.

[79] Egli S, Ayer F, Peter M, Eilmann B, Rigling A (2010). Is forest mushroom productivity driven by tree growth? Results from a thinning experiment. Ann Forest Sci.

[80] Tomao A, Bonet JA, de Aragón JM, de Miguel S (2017). Is silviculture able to enhance wild forest mushroom resources? Current knowledge and future perspectives. Forest Ecol Manage.

[81] Dickie IA, Reich PB (2005). Ectomycorrhizal fungal communities at forest edges. J Ecol.

[82] Dickie IA, Dentinger BTM, Avis PG, McLaughlin DJ, Reich PB (2009). Ectomycorrhizal fungal communities of oak savanna are distinct from forest communities. Mycologia.

[83] Żółciak, A. Inokulacja pniaków liściastych grzybnią boczniaka ostrygowatego [Pleurotus ostreatus] jako biologiczna metoda zabezpieczania przed opieńkową zgnilizną korzeni. Prace Instytutu Badawczego Leśnictwa. 2002;Seria A, (4 [944-947]), 5-19.

[84] Schlumpf E. Sollen unsere Pilze aussterben. Schweizerische Zeitschrift Pilzkunde. Benteli Verlag. 1976;54:101–5.

[85] Jansen E, de Wit T (1978). Veranderingen in de verspreiding van de Cantharel in Nederland. Coolia.

[86] Jaenike J (1991). Mass Extinction of European Fungi. Tree.

[87] Cherfas J (1991). Disappearing mushrooms: another mass extinction?. Science.

[88] Arnolds E (1988). The changing macromycete flora in the Netherlands. Transact Br Mycol Soc.

[89] Arnolds E (1991). Decline of ectomycorrhizal fungi in Europe. Agri Ecosyst Environ.

[90] Arnolds E (1995). Conservation and management of natural populations of edible fungi. Can J Bot.

[91] Molnár Z. Classification of pasture habitats by Hungarian herders in a steppe landscape (Hungary). J Ethnobiol Ethnomed. 2012;8(1):28. 10.1186/1746-4269-8-28. 10.1186/1746-4269-8-28 PMC3533854 22853549

[92] Kundzewicz, Z. W., & Matczak, P. Climate change regional review. Wiley Interdisciplinary Reviews: Climate Change, 2012;3(4), 297-311. doi: 10.1002/wcc.175

[93] Somorowska U (2017). Soil water storage in Poland over the years 2000-2015 in response to precipitation variability as retrieved from GLDAS Noah simulations. Geographia Polonica.

[94] Wiklund K, Nilsson LO, Jacobsson S (1995). Effect of irrigation, fertilization, and artificial drought on basidioma production in a Norway spruce stand. Can J Bot.

[95] Ogaya R, Peñuelas J (2005). Decreased mushroom production in a holm oak forest in response to an experimental drought. Forestry.

[96] De Aragón JM, Bonet JA, Fischer CR, Colinas C. Productivity of ectomycorrhizal and selected edible saprotrophic fungi in pine forests of the pre-Pyrenees mountains, Spain: predictive equations for forest management of mycological resources. Forest Ecol Manage. 2007;252(1-3):239–56. 10.1016/j.foreco.2007.06.040.

[97] Wilkins WH, Harris GCM (1946). The ecology of the larger fungi V. An investigation into the influence of rainfall and temperature on seasonal production of fungi in a beechwood and a pinewood. Ann Appl Biol.

[98] Dahlberg A (1991). Ectomycorrhiza in coniferous forest: structure and dynamics of populations and communities.

[99] Gange AC, Gange EG, Sparks TH, Boddy L (2007). Rapid and recent changes in fungal fruiting patterns. Science.

[100] Schär C, Vidale PL, Lüthi D, Frei C, Häberli C, Liniger MA, Appenzeller C (2004). The role of increasing temperature variability in European summer heatwaves. Nature.

[101] Kręgiel B, Jarosińska E (2009). Obecny stan monitoringu zjawiska suszy w Polsce i na świecie. Czasopismo Techn Środowisko.

[102] Egli S, Peter M, Buser C, Stahel W, Ayer F (2006). Mushroom picking does not impair future harvests–results of a long-term study in Switzerland. Biol Conserv.

[103] Frey B, Kremer J, Rüdt A, Sciacca S, Matthies D, Lüscher P (2009). Compaction of forest soils with heavy logging machinery affects soil bacterial community structure. Eur J Soil Biol.

[104] Vitousek PM (1994). Beyond global warming: ecology and global change. Ecology.

[105] Kottke I, Qian XM, Pritsch K, Haug I, Oberwinkler F (1998). Xerocomus badius–Picea abies, an ectomycorrhiza of high activity and element storage capacity in acidic soil. Mycorrhiza.

[106] Rosinger C, Sandén H, Matthews B, Mayer M, Godbold D (2018). Patterns in ectomycorrhizal diversity, community composition, and exploration types in European beech, pine, and spruce forests. Forests.

[107] Clemmensen KE, Michelsen A, Jonasson S, Shaver GR (2006). Increased ectomycorrhizal fungal abundance after long-term fertilization and warming of two arctic tundra ecosystems. New Phytologist.

[108] Morgado LN, Semenova TA, Welker JM, Walker MD, Smets E, Geml J (2015). Summer temperature increase has distinct effects on the ectomycorrhizal fungal communities of moist tussock and dry tundra in Arctic Alaska. Global Change Biol.

[109] Fernandez CW, Nguyen NH, Stefanski A, Han Y, Hobbie SE, Montgomery RA, Kennedy PG (2017). Ectomycorrhizal fungal response to warming is linked to poor host performance at the boreal-temperate ecotone. Global Change Biol.

[110] Lamhamedi MS, Bernier PY, André-Fortin J (1992). Hydraulic conductance and soil water potential at the soil–root interface of Pinus pinaster seedlings inoculated with different dikaryons of Pisolithus sp. Tree Physiol.

[111] Risbeth J (1972). The production of rhizomorphs by Armillaria mellea from stumps. Eur. J. For. Path.

[112] Szwed M, Karg G, Pińskwar I, Radziejewski M, Graczyk D, Kędziora A, Kundzewicz ZW (2010). Climate change and its effect on agriculture, water resources and human health sectors in Poland. Nat Hazards Earth Syst Sci.

[113] International Society of Ethnobiology Code of Ethics (with 2008 additions). 2013; Available from: http://ethnobiology.net/code-of-ethics [Accessed on 2018 Aug 17]

[114] American Anthropological Association Code of Ethics. 2009; Available from: http://s3.amazonaws.com/rdcms-aaa/files/production/public/FileDownloads/pdfs/issues/policy-advocacy/upload/AAA-Ethics-Code2009.pdf [Accessed on 2018 Aug 17]

